# Developmental change of brain volume in Rett syndrome in Taiwan

**DOI:** 10.1186/s11689-024-09549-6

**Published:** 2024-07-03

**Authors:** Tz-Yun Jan, Lee-Chin Wong, Chia-Jui Hsu, Chien-Feng Judith Huang, Steven Shinn-Forng Peng, Wen-Yih Isaac Tseng, Wang-Tso Lee

**Affiliations:** 1 Graduate Institute of Brain and Mind Sciences, National Taiwan University College of Medicine, Taipei, Taiwan; 2 Institute of Medical Device and Imaging, National Taiwan University College of Medicine, Taipei, Taiwan; 3 Institute of Biomedical Engineering, National Taiwan University College of Medicine, Taipei, Taiwan; 4 Department of Pediatrics, Cathay General Hospital, Taipei, Taiwan; 5 Department of Pediatrics, National Taiwan University Hospital Hsin-Chu Branch, Hsin-Chu, Taiwan; 6 Department of Medical Imaging, National Taiwan University Hospital and National Taiwan University College of Medicine, Taipei, Taiwan; 7 Molecular Imaging Center, National Taiwan University, Taipei, Taiwan; 8 Department of Pediatrics, National Taiwan University Hospital and National Taiwan University College of Medicine, 8, Chung-Shan South Road, Taipei, 100 Taiwan

**Keywords:** Rett syndrome, Magnetic resonance image, Brain volume, Total intracranial volume, Developmental trajectories, Gray matter volume

## Abstract

**Objective:**

Rett syndrome (RTT) is characterized by neurological regression. This pioneering study investigated the effect of age on brain volume reduction by analyzing magnetic resonance imaging findings in participants with RTT, ranging from toddlers to adults.

**Methods:**

Functional evaluation and neuroimaging were performed. All scans were acquired using a Siemens Tim Trio 3 T scanner with a 32-channel head coil.

**Results:**

The total intracranial volume and cerebral white matter volume significantly increased with age in the control group compared with that in the RTT group (*p* < 0.05). Cortical gray matter volume reduction in the RTT group continued to increase in bilateral parietal lobes and left occipital lobes (*p* < 0.05). The differences in cortical gray matter volume between typically developing brain and RTT-affected brain may tend to continuously increase until adulthood in both temporal lobes although not significant after correction for multiple comparison.

**Conclusions:**

A significant reduction in brain volume was observed in the RTT group. Cortical gray matter volume in the RTT group continued to reduce in bilateral parietal lobes and left occipital lobes. These results provide a baseline for future studies on the effect of RTT treatment and related neuroscience research.

**Supplementary Information:**

The online version contains supplementary material available at 10.1186/s11689-024-09549-6.

## Introduction

Rett syndrome (RTT), a rare neurodevelopmental disease, may present with an exclusive trajectory change in clinical phenotypes. The 2010 revision of diagnostic criteria defined main, supporting criteria and exclusion criteria, reflecting the neurological progression of the disease [38]. Although individuals with RTT may have a small head circumference, they generally develop normally till the age of 6–18 months [24], after which they exhibit progressive problems in motor coordination, learning, memory, communication, and other neurodevelopment. Typical patients with RTT may present with a partial or complete loss of acquired hand skills, followed by stereotypical hand movements. Moreover, communication and mobility are impaired or absent, and most patients require assistance in their daily life [23, 24, 38]. Therefore, the clinical presentations of individuals with RTT may be classified into 4 stages based on the progression of the disease [38]. In Stage I of RTT, there is an early onset of developmental stagnation, which begins at approximately 6 months to 1.5 years of age, and microcephaly and loss of language gradually begin to appear. In Stage 2, developmental regression occurs at 1–4 years of age, and the affected individuals lose acquired skills in language and behavior. In Stage 3, which develops at approximately 4–7 years of age, some individuals with RTT may regain certain abilities such as communication or language skills. In Stage 4, there is further deterioration of motor skills, and individuals may become nonambulatory and have more severe scoliosis.

The methyl-CpG-binding protein 2 (*MECP2*) gene, a crucial gene that causes RTT, was first identified as a nuclear protein with a transcriptional repressor role. It is expressed in very early neuronal progenitors and has a well-known function in neurodevelopment. MECP2 plays a crucial role in neuronal maturation, terminal differentiation, and synaptic formation [33]. The expression of MECP2 is highest in the cerebral cortex and cerebellum, and the expression can continue into adulthood and is also much higher in adult brain than that in the infantile stage. Therefore, it can also affect the development of brain and its function in adulthood [13, 21, 35]. MECP2, as a key epigenetic modulator in the brain, is involved in promoting embryonic development and stem cell differentiation and therefore plays a critical role in human disease.

The normal development of the brain is associated with age and follows a specific pattern of cortical development and functions [30]. The intracranial space increases dramatically after birth but increases very slightly after the first decade [42]. There are increases in the cortical area, decreases in cortical thickness, and variable changes in cortical volume in different regions. There are also increases in the volumes of cerebral subcortical structures, such as deep nuclei, and the cerebellum, when the expression of MECP2 increases [8]. During childhood and adolescence stages, dynamic changes occur in different brain structures, exhibiting continuous age-related decreases in the volumes of the frontal cortex, thalamus, and nucleus accumbens, and the cerebral white matter volume also increases during childhood and early adulthood [8, 42]. The developing gray matter also reaches the peak of growth in later childhood, after which it decreases linearly with age [11]. Nevertheless, there has been no study focusing on the longitudinal age-related change in brain volume using magnetic resonance imaging (MRI). The only study on age–brain volume correlation in RTT demonstrated a decrease in the size of the cerebellum with age, but there was no evidence of a progressive decrease in the volume of the cerebral cortex with age [37]. Moreover, the majority of studies on brain volume changes in RTT focused on children and adolescents, when most individuals are in stages 2–3, but not on adults, when the expression of MECP2 is higher than that in childhood, and the individuals are in stage 4 [6, 9, 20, 37, 40]. The clinical stages may also reflect the brain development of these individuals with RTT. Therefore, the effect of *MECP2* on the brain in different stages of RTT has been less investigated in the past, especially regarding the difference in younger age and older age when the staging is different.

MRI is a widespread and noninvasive neuroimaging tool for evaluating brain structure and function and for investigating the association between neural activities and clinical events. An MRI study using quantitative measurement revealed a widespread decrease in gray matter volume but a relative preservation of the posterior occipital region in RTT [37]. However, Reiss et al. [40] published the first volumetric MRI study that computed the brain by classifying it into 16 regions and suggested that the largest decrease in cortical gray matter volume was in the frontal region. There was also greater loss of gray matter compared with white matter. Significant reductions were also observed in the parietal and superior parieto-occipital region and basal ganglia in subcortical areas [40]. Another study that used the Talairach data processing and voxel-based morphometry recruited patients aged 5–12 years demonstrated a reduction in the volume of the frontal lobe and dorsal parietal region [9]. Furthermore, two small cohort studies on adolescent and adult patients demonstrated a comparative reduction in brain volume in the frontal lobe, temporal lobe, and cerebellum [6, 20]. Although previous studies have investigated the change in gray matter volume in RTT, the effect of age-related changes on cortical gray matter was rarely reported [6, 20].

This study focused on region-dependent brain volume changes in the cortical gray matter in individuals of younger age and older age with RTT. We explored the increasing trend of intracranial volume and cortical gray matter volume in individuals with RTT compared with that in neurotypical individuals.

## Methods

### Participants

A total of 28 patients with RTT aged 2–27 years were recruited in this study. All participants were enrolled and followed up at the Joint Clinic for RTT in National Taiwan University Hospital (NTUH), Taiwan. RTT was diagnosed based on the revised diagnostic criteria [38] by a senior pediatric neurologist (Lee W.T.). The control group included 32 sex-matched participants whose ages were paired with individuals with RTT within 6 months of range. Only one male subject was enrolled in each group (Table 1). All participants in the control group completed an interview by pediatric neurologists, which included detailed developmental history, birth history, family history, and neurological examinations. Participants in the control group displayed typical behavioral and neurological development. Individuals with any neurological diseases (e.g., psychiatric diagnoses, history of neurological impairment, neuropsychiatric conditions, and clinical evidence of a genetic disorder) were excluded. Remaining subjects were suitable for participation in the MRI study. To avoid motion-related artifacts, individuals with RTT and younger neurotypical individuals (aged ≤ 4 years) were routinely sedated using chloral hydrate before performing MRI. Older neurotypical individuals were also sedated if there were motion artifacts after repeating scanning. The 28 patients with RTT were classified into a younger group (aged < 10 years) and an older group (aged > 10 years) (Table 1), and the differences between the two age groups were compared in this study.

**Table 1:** Demographic characteristic and clinical status of patients with RTT and controls

		Younger group		Older group		*p* value
Characteristic		RTT (*n* = 12)	Controls (*n* = 12)	RTT (*n* = 16)	Controls (*n* = 20)	
Age, mean ± SD		5.46 ± 2.30	5.694 ± 1.80	19.05 ± 4.95	19.813 ± 5.46	NS
Sex (M/F)		0/12	0/12	1/15	1/19	
Gene mutation: *MECP2*/NA		11/1	-	14/2	-	
Mean years of rehabilitation or education		3.118	2.1	14.56	15.6	
Handedness (R/L/NA)		2/0/10	12/0/0	3/0/13	20/0/0	
Speech: words/mute		5/7	12/0	2/14	20/0	
Rett syndrome stage, n (%)		Younger group		Older group		
Stage 2		8 (66.6)		0		
Stage 3		2 (16.7)		12 (75)		
Stage 4		2 (16.7)		4 (25)		
RSBQ		43.17 ± 13.51		43.06 ± 12.1		0.963
RSSS		9.17 ± 3.251		11.76 ± 3.2		0.024
Peabody Developmental Motor Scales -II						
Stationary	Raw Score	32.583 ± 6.61		30.63 ± 7.09		0.401
	Age Equivalent	13.08 ± 9.93		9.25 ± 4.27		0.118
Locomotion	Raw Score	62.00 ± 33.77		57.00 ± 30.43		0.605
	Age Equivalent	12.42 ± 7.47		11.13 ± 6.04		0.525
Object Manipulation	Raw Score	4.167 ± 8.99		1.25 ± 3.55		0.116
	Age Equivalent	6.67 ± 11.43		2.75 ± 6.15		0.175
Grasping	Raw Score	18.50 ± 17.04		17.44 ± 15.49		0.796
	Age Equivalent	9.00 ± 11.71		6.19 ± 9.68		0.16
Visual-Motor	Raw Score	34.50 ± 29.09		29.50 ± 27.73		0.344
	Age Equivalent	7.67 ± 6.83		6.81 ± 7.67		0.314

*RTT* Rett syndrome, *NA* not applicable, *NS* non-significant

Parents and caregivers were requested to complete developmental and clinical questionnaires. The motor function of all participants with RTT was evaluated using the Peabody Developmental Motor Scales-second edition (PDMS-2). All evaluations were performed by the same occupational therapist to ensure quality and consistency. This study was approved by the institutional review board of NTUH in Taipei, Taiwan (201510011RINC). The request to complete the questionnaires and participate in the imaging studies was approved by the internal review board.

### Image data acquisition and processing

All scans were acquired using a Siemens Tim Trio 3 T scanner with a 32-channel head coil at NTUH. Because potential motion artifacts and general reconstruction issues may occur in the MRI scans of nonsedated individuals with RTT with severe disability, all patients with RTT or younger neurotypical individuals were sedated during MRI in this study, and the heart rate and blood O_2_ saturation were continuously monitored using pulse oximetry.

The MRI protocol consisted of sagittal T1-weighted and axial fast spin-echo T2-weighted imaging. High-resolution T1-weighted imaging was performed using 3D magnetization-prepared rapid gradient-echo (MPRAGE), which was further used for anatomical reference and calculations of gray matter volume. Transverse sections were obtained parallel to the anterior and posterior commissure line with a thickness of 1.0 mm^3^. The total number of slices per slab was 208 with repetition time (TR) = 2000 ms, echo time (TE) = 2.98 ms, flip angle = 9°, and field of view (FOV) read = 256 × 192 mm^2^. The fast spin-echo sequence was acquired from 56 contiguous axial T2-weighted images with TR = 9650 ms, TE = 103 ms, FOV = 200 × 200 mm^2^, and slice thickness = 2.5 mm.

#### Protocol

Imaging processing and analysis were completed in the following steps. All scans passed a successful quality control test for gross structural abnormalities before data processing. The actual coordinates were adjusted using displayed methods on the Statistical Parametric Mapping 12 software package (http://www.fil.ion.ucl.ac.uk/spm/software/spm12/) that was run through in MATLAB 2016 (Mathworks, USA) to avoid artifacts, poor directionality, and insufficient image quality.

For calculating the gray matter volume, the FreeSurfer software package (v.6.0.0–64 bit; Martinos Center for Biomedical Imaging, Boston, MA, USA) was used, which created a three-dimensional model of the cortical surface and cortical thickness along with area measurements. The gray matter volume in this study indicated the cortical gray volume and did not include the subcortical gray matter and cerebellum. Segmentation of the gray and white matter volume was performed using the FreeSurfer “recon-all” pipeline (http://surfer.nmr.mgh.harvard.edu) based on intensities and continuity of information from the full-head image to establish representations of the boundary of the gray/white matter (surface) and the pial surface. For reconstruction and estimation, motion correction for better segmentation in the original imaging was performed before the procedure to ensure the accuracy of T1-weighted images. Although the FreeSurfer software includes motion correction, the removal of nonbrain tissue, and automated intensity normalization for surface- and intensity-based segmentation of the cortex, FreeSurfer segmentations would be visually checked and manually corrected or excluded for all individuals by a neuroradiologist with experience in segmentation after completing the scans. If there was incorrect segmentation, the neuroradiologist would perform manual correction before further processing. To reduce motion artifacts, all participants would be comforted for at least 30 min before scanning. For participants with RTT or younger neurotypical individuals, we would continue to comfort them after sedation and lying on the MRI machine. After scanning, for those with motion artifacts, which resulted in incorrect segmentations, we would repeat all scans on the same day or another day to obtain better images. For some individuals, the scans had to be repeated at least two to three times to acquire better images for analysis. For almost all healthy control individuals, the scans can be completed at the first time because no motion artifacts were observed and there was good brain volume segmentation. Although all participants with RTT were sedated during MRI processing, there was also no significant difference in brain volume segmentation for sedative (individuals with RTT) and nonsedative individuals (healthy controls) in our analysis after scanning, despite the failure rate being much higher in individuals with RTT due to motion artifacts.

The brain tissue was automatically transformed from the topological surface to the Talairach space. Volumetric structures were segmented for the tessellation of the gray matter–white matter boundary [14, 17]. Subsequently, the intensity gradients, automated topology, and deformed surface corrections were applied to optimize the locations of the gray/white and gray/cerebrospinal fluid borders for the greatest shift in intensity [15, 16]. The automatic labeling method provided similar results as those of manual labeling in previous studies and demonstrated low reproducibility errors and high precision [15, 25].

The intracranial volume and gray matter and white matter volumes were acquired from the abovementioned procedure. Moreover, considering the feasibilities, consistency, and accuracy that were required for investigating the gray matter volume, Desikan–Killiany–Tourville (DKT) cortical labeling atlases (rh.aparc.DKTatlas.stats/ lh.aparc.DKTatlas.stats) were selected for volumetric data analysis and manual correction [12] to investigate local changes in the gray matter. Of 31 cortical regions in DKT atlases, 24 were selected and subdivided into four lobar groups, and the differences between the two age groups were compared. The volumes of the frontal lobe included those of the superior frontal gyrus, middle frontal gyrus (rostral part and caudal part), inferior frontal gyrus (pars orbitalis, pars opercularis, and pars triangularis), precentral gyrus, and orbitofrontal gyrus (medial and lateral division). The parietal lobe included the superior parietal lobule, inferior parietal lobule, supramarginal gyrus, postcentral gyrus, paracentral gyrus, and precuneus. The temporal lobe included the superior temporal gyrus, middle temporal gyrus, inferior temporal gyrus, transverse temporal gyrus, and fusiform gyrus. The occipital lobes encompassed the lingual gyrus, pericalcarine cortex, cuneus cortex, and lateral occipital cortex [28].

### Clinical assessments

PDMS-2 is a reliable and valid tool used to evaluate motor functions [10], it was administered to evaluate the gross and fine motor skills objectively [18, 32]. PDMS-2 is a motor development program comprising the following six subtests: reflexes, stationary, locomotion, object manipulation, grasping, and visual–motor integration. The items of reflexes were excluded because reflexes are inhibited at the cortical level in normal development. For each subtest task, performance criteria were specified and scored on a 3-point scale, from 0 to 2, where 0 indicates bad and 2 implies mastered. For administration, a basal level was determined when a score of 2 was obtained on three consecutive items, and a ceiling level was determined when a score of 0 was obtained on three consecutive items.

The Rett Syndrome Behavior Questionnaire (RSBQ) evaluates typical pathological behaviors and clinical symptoms in individuals with RTT [5, 26, 36]. A total of 45 items were categorized into the following eight domains/subscales: (1) general mood, (2) breathing problems, (3) body rocking and expressionless face, (4) hand behaviors, (5) repetitive face movements, (6) nighttime behaviors, (7) fear/anxiety, and (8) walking/standing. Each item was scored on a Likert scale of 0–2 based on how well the description fitted the patient’s behavior.

The Rett Syndrome Severity Scale (RSSS) includes the following seven domains: (1) frequency and manageability of seizures, (2) respiratory irregularities, (3) scoliosis, (4) ability to walk, (5) hand use, (6) speech, and (7) sleep. Each item was scored on a Likert scale of 0–3 based on the probability that the description portrays the severity of symptom. Total scores were then categorized into three degrees, viz., mild (0–7), moderate (8–14), and severe (15–21) [27, 34].

### Statistical analysis

All data were expressed as number or mean ± standard deviation. The difference in the scores of RSBQ and RSSS and the performance differences in the five subtests of PDMS-2 among younger and older individuals with RTT were compared using independent t-tests.

Imaging data were analyzed for the total intracranial volume (TIV), total cortical gray matter volume, and cerebral white matter volume. An independent t-test was used to analyze the differences in intracranial volume, cerebral white matter volume, cortical gray matter volume, and cortical gray matter volume in four cerebral lobes between the RTT group and control group. To further analyze the lobe differences between the RTT group and control group, the one-way analysis of covariance (one-way ANCOVA) was used and adjusted by the covariate (TIV). Moreover, to determine the association between brain volume and age changes, the volume changes were incorporated into a linear regression model with age and age squared for developmental trajectories as follows:

$$\text{The volume of each cerebral lobe}=\text{b}0+\text{ b}1\left(\text{group}\right)+\text{b}2\left(\text{age}\right)+\text{b}3\left(\text{age squared}\right)+\text{b}4\left({\text{group}}^{*}\text{age}\right)+\text{b}5\left({\text{group}}^{*}\text{age squared}\right).$$

In the analysis, we investigated the effect of age and group and also the interaction between group and age. Finally, the general linear model was applied to investigate group differences in age-related structural changes. The linear model represented the relationships among cortical gray matter volume in four cerebral lobes and the following input variables: age, age squared, group, age-by-group interaction, and age-squared-by-group interaction. These variables were corrected using Benjamini–Hochberg false discovery rate (FDR) for multiple comparisons in q-value. These relationships were compared with the average values and slopes of both groups. A Levene’s test was used to confirm the homogeneity of the variance of both RTT and control groups before implementing the one-way ANCOVA and general linear model.

The Levene’s test revealed a greater *p* value than the critical value (0.05), which supported the hypothesis of the Levene’s test that the variances were equal. The Bonferroni correction was also applied to minimize their effects on the study variables.

All statistical analyses were conducted using IBM SPSS Statistics 20 (SPSS Inc., Chicago, Illinois, USA). A *p* value of < 0.05 was considered statistically significant.

### Community involvement

The families of patients with RTT in Taiwan and the Rett Syndrome Association in Taiwan were actively involved and supported throughout this study process, and they participated in interviews, surveys, and other data collection. We anticipate that they will be aware of the effect of MECP2 variants on brain development, especially the long-term effect on brain development.

## Results

### Demographic data

Data from 28 patients with RTT were obtained and classified into a younger group (< 10 years) and an older group (> 10 years) (Table 1). In the younger group, all individuals were in stages 2–4; in the older group, the individuals were in stages 3–4 (Table 1). In both groups, there were no significant differences in age between individuals in the RTT group and control group. A mutation in *MECP2* was detected in 25 of 28 (89.2%) patients with typical RTT; this percentage was higher than that reported in a previous cross-national survey [43]. Only three patients with typical RTT did not harbor gene mutations. Due to the regression of hand functions, 23 patients with RTT did not have dominant handedness, whereas the remaining individuals with RTT and all neurotypical individuals had right-hand dominance. In the younger RTT group, 8 (66.6%) participants were in stage 2, and the remaining participants regressed to stage 3 (16.7%) or stage 4 (16.7%). In contrast, 12 participants (75%) in the older group were in stage 3, and the other 4 individuals were in stage 4 (25%). Seven patients in this study could speak at a word level (less than five words), of whom two were in the older group, aged 17 and 21 years, respectively, and the others were females in the younger group.

### Behavioral results in neuromotor development and clinical presentations

Regarding clinical manifestations (Table 1), RSBQ revealed no difference between the two RTT groups (younger group: M = 43.17 ± 13.51; older group: M = 43.06 ± 12.1, *p* = 0.963). Although the raw scores of motor development subtests in PDMS-2 were higher in the younger group, there was no significant difference (Table 1). The categories of severity showed a moderate level in both age groups of the RTT group. However, the older RTT group exhibited significantly higher severity than the younger RTT group on RSSS (*p* = 0.024) (Table 1).

### Changes in TIV, cortical gray matter volume and cerebral white matter volume

**Fig. 1:**
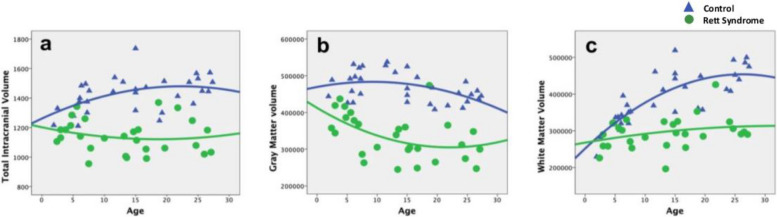
The difference of developmental trajectories in (**A**) total intracranial volume (cm^3^), **B** cortical gray matter volume (mm^3^), and **C** cerebral white matter (mm^3^) from 28 individuals with RTT (triangle) and 32 controls (circle), ages 2 to 27 years

Significant differences were observed between the RTT group and control group in TIV (*p* < 0.001), cortical gray matter volume (*p* < 0.001), and cerebral white matter volume (*p* < 0.001) (Supple. Table 1) (Fig. 1). We evaluated the developmental change using the regression model to examine the development of brain structures in individuals with RTT compared with that in neurotypical individuals. A significant interaction effect (age-by-group and age-squared-by-group) was found in TIV and cerebral white matter volume in the linear regression model (TIV: B = 8.316, *p* = 0.022; WM: B = 4961.36, *p* = 0.001) and the quadratic regression model (TIV: B = 0.242, *p* = 0.049; WM: B = 156.841, *p* = 0.002) (Supple. Table 2). Results also revealed significant differences between the two groups with increasing age. Compared with that in the RTT group, TIV and cerebral white matter volume increased on average by 8.316 cm^3^ and 4961.36 mm^3^, respectively, in the control group. In contrast, the cortical gray matter volume significantly decreased with increasing age (B = − 3408.171, *p* = 0.003) in both group (Supple. Table 2), but there was no difference in both groups.

### Different reductions of regional brain volume in the younger and older groups

Considering the significant difference in the total cortical gray matter volume between the RTT and control groups, we next investigated the possible differences in different cerebral lobes. We found significant decreases in cortical gray matter volume in all four cerebral lobes in the RTT group (*p* < 0.001) (Supple. Table 3). We then clarified the effect of age on the decrease of volume in both RTT and control groups using one-way ANCOVA. This method was based on the linearly independent pairwise comparisons among the estimated marginal mean values with TIV as the covariate in the model. In the younger group (Table 2), the brain volume of individuals in the RTT group was significantly lower than that in the control group in the right frontal lobes (*p* = 0.012), left frontal lobes (*p* = 0.007), right parietal lobes (*p* = 0.006), left parietal lobes (*p* = 0.0019), and right occipital lobe (*p* = 0.005). However, no significant differences were observed in the volume of the temporal lobes. In contrast, a significant decrease was observed in the brain volumes of individuals with RTT in the older group across the right frontal lobes (*p* = 0.029), parietal lobes (R/L: *p* = 0.005/0.01), temporal lobes (R/L: *p* = 0.011/0.047), and occipital lobes (R/L: *p* = 0.025/0.019) (Table 3). This result indicated the involvement of different lobes in the entire brain in individuals with RTT in the older group. Among younger (aged ≤ 10 years) participants, the volume of temporal lobes in participant with RTT was not significantly different from that in controls (R/L: *p* = 0.426/0.176). Interestingly, among older participants (aged > 10 years), the volume of temporal lobes in participants with RTT was significantly smaller than that in controls (R/L: *p* = 0.011/0.047).

**Table 2:** Regional difference of cortical gray matter volume in young group of Rett syndrome compared to healthy control (one-way ANCOVA, TIV as covariate)

	**Control group (mm**^**3**^**)**	**Rett syndrome group (mm**^**3**^**)**	**F**	***p* value**
R_Frontal lobe	82332.37 ± 2433.54	71493.21 ± 2433.54	7.464	0.012^*^
L_Frontal lobe	82689.15 ± 2429.57	70934.18 ± 2429.57	8.807	0.007^**^
R_Parietal lobe	67964.16 ± 2247.62	56629.49 ± 2247.62	9.568	0.006^**^
L_Parietal lobe	65181.14 ± 2475.15	54919.02 ± 2475.15	6.467	0.019^*^
R_Temporal lobe	48549.87 ± 1588.12	46447.70 ± 1588.12	0.659	0.426
L_Temporal lobe	48948.15 ± 1387.48	45777.17 ± 1387.48	1.965	0.176
R_Occipital lobe	26927.53 ± 2687.96	20486.07 ± 3754.82	8.448	0.005^**^
L_Occipital lobe	26020.31 ± 1029.08	24199.43 ± 1029.08	1.178	0.290

Adjustment for multiple comparisons: Bonferroni

*TIV* Total intracranial volume

Estimated marginal means ± standardized error; ^*^p < 0.05; ^**^p < 0.01

**Table 3:** Regional difference of cortical gray matter volume in old group of Rett syndrome compared to healthy control (one-way ANCOVA, TIV as covariate)

	**Control group (mm**^**3**^**)**	**Rett syndrome group (mm**^**3**^**)**	**F**	***p* value**
R_Frontal lobe	79083.70 ± 2788.80	66847.81 ± 3312.754	5.204	0.029^*^
L_Frontal lobe	77978.06 ± 2711.88	68037.36 ± 3221.38	3.632	0.065
R_Parietal lobe	61225.54 ± 2182.81	48515.38 ± 2592.92	9.165	0.005^**^
L_Parietal lobe	59560.00 ± 2175.99	48185.48 ± 2584.81	7.386	0.010^*^
R_Temporal lobe	50112.58 ± 1605.48	41799.27 ± 1907.12	7.248	0.011^*^
L_Temporal lobe	49472.04 ± 1650.0	42937.19 ± 1960.00	4.240	0.047^*^
R_Occipital lobe	24759.61 ± 854.45	20903.61 ± 1014.99	5.505	0.025^*^
L_Occipital lobe	24198.07 ± 780.75	20505.40 ± 927.44	6.047	0.019^*^

Adjustment for multiple comparisons: Bonferroni

*TIV* total intracranial volume

Estimated marginal means ± standardized error; ^*^p < 0.05; ^**^p < 0.01

**Fig. 2:**
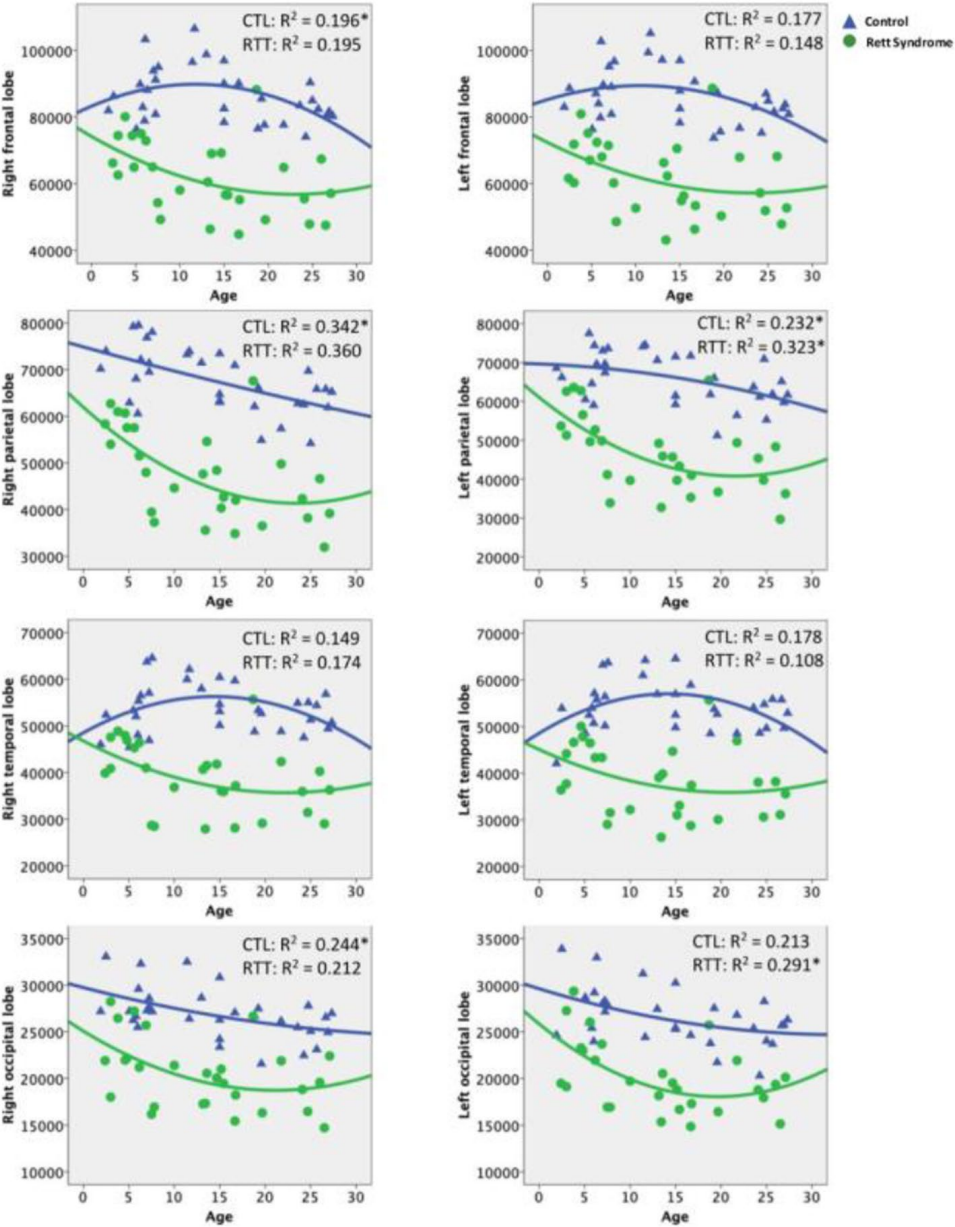
The decreasing trend showed in brain development of RTT in cortical gray matter. The triangle indicated controls; the dot indicated RTT group. In four cerebral lobes, the development of gray matter in individual with RTT start with a decline in volume until it reached a plateau around adult period. In four cerebral lobes, only the development of bilateral temporal lobes in individuals with RTT showed significant decrease by age from the control group in general linear model

We applied the regression model to investigate the age-related changes in different lobes in individuals with RTT. Results demonstrated age-related changes in gray matter volume in bilateral parietal lobes and the left occipital lobe in individuals with RTT, showing significantly greater reduction in terms of age (*p* < 0.05, Table 4); however, this was not statistically significant in terms of age squared. This result indicated a significant linear correlation between age and gray matter volume rather than a curvilinear correlation. The development of cortical gray matter in the parietal lobes of individuals with RTT started with a decline in volume until it reached a plateau at the age of 23 years in the right parietal lobe and at the age of 21 years in the left parietal lobe (Fig. 2, Table 4).

**Table 4:** Developmental curve for bilateral cerebral lobes in RTT and control groups

	**Right frontal lobe**		**Right parietal lobe**		**Right temporal lobe**		**Right occipital lobe**	
	**CTL**	**RTT**	**CTL**	**RTT**	**CTL**	**RTT**	**CTL**	**RTT**
R^2^	0.196^*^	0.195	0.342*	0.360^*^	0.149	0.174	0.244^*^	0.212
(Constant)	83276.216^**^	74186.016^**^	74912.824^**^	61965.748^**^	48575.914^**^	46791.692^**^	29805.800^**^	25084.060^**^
Age	1122.056	-1516.199	-543.479	-1756.006^*^	1068.443^*^	-1001.248	-263.426	-600.970
Age^2^	-47.824	33.090	2.250	37.425	-37.108^*^	22.575	3.426	14.216
	**Left frontal lobe**		**Left parietal lobe**		**Left temporal lobe**		**Left occipital lobe**	
	**CTL**	**RTT**	**CTL**	**RTT**	**CTL**	**RTT**	**CTL**	**RTT**
R^2^	0.177	0.148	0.232^*^	0.323^*^	0.178	0.108	0.213^*^	0.291^*^
(Constant)	85270.561^**^	72407.054^**^	69689.138^**^	61033.937^**^	48642.587^**^	45077.847^**^	29649.371^**^	25846.649^**^
Age	792.224	-1307.466	-100.478	-1871.639^*^	1172.857	-880.850	-304.951	-794.188^*^
Age^2^	-37.716	28.182	-9.104	43.270	-41.208	21.091	4.739	20.234

*CTL* control group, *RTT* Rett syndrome group

^*^p < 0.05; ^**^p < 0.01

### Relationship among age, groups and cortical gray matter volume in RTT and control groups

We applied a general linear model to further compare the age-related gray matter volume difference in different cerebral lobes between the RTT and control groups (Table 5). We found a significant difference in all cerebral lobes in the RTT and control groups, consistent with the findings shown in Supple. Table 3. Age tended to be associated with the reduction of the cortical gray matter volume in the bilateral parietal lobes and left occipital lobe in individuals with RTT, which was consistent with results shown in in Table 4. Although the effects of age-by-group and age-squared-by-group interactions on the cortical gray matter volume were observed in the bilateral temporal lobes (age * group R/L: *p* = 0.023/0.030; age squared * group R/L:* p* = 0.047/0.046) (Table 5), they became insignificant after FDR correction. This results suggests that the cortical gray matter volume differences between typically developing brain and RTT-affected brain tend to continuously increase until adulthood in both temporal lobes although not significant after FDR correction.

**Table 5:** The results of general linear model showing the group interaction with age

	**Right frontal Lobe**			**Right parietal lobe**			**Right temporal lobe**			**Right occipital lobe**		
	R^2^	0.697		R^2^	0.745		R^2^	0.617		R^2^	0.617	
	B	p	q	B	p	q	B	p	q	B	p	q
Intercept	74186.016	< 0.001	0.006	61965.748	< 0.001	0.006	46791.692	< 0.001	0.006	25084.06	< 0.001	0.006
Group	9090.201	0.274	0.411	12947.076	0.044	0.088	1784.222	0.745	0.745	4721.74	0.086	0.172
Age	-1516.199	0.11	0.132	-1756.006	0.017	0.051	-1001.248	0.112	0.168	-600.97	0.055	0.165
Age^2^	33.09	0.3	0.300	37.425	0.128	0.192	22.575	0.287	0.344	14.216	0.178	0.267
Group^*^ Age	2638.255	0.054	0.162	1212.528	0.242	0.290	2069.69	0.023	0.069	337.543	0.446	0.535
Group^*^ Age^2^	-80.914	0.074	0.148	-35.175	0.305	0.305	-59.683	0.047	0.094	-10.79	0.462	0.462
	**Left frontal Lobe**			**Left parietal lobe**			**Left temporal lobe**			**Left occipital lobe**		
	R^2^	0.684		R^2^	0.708		R^2^	0.663		R^2^	0.623	
	B	p	q	B	p	q	B	p	q	B	p	q
Intercept	72407.054	< 0.001	0.006	61033.937	< 0.001	0.006	45077.847	< 0.001	0.006	25846.649	< 0.001	0.006
Group	12922.762	0.0131	0.039	8738.522	0.187	0.187	3481.419	0.544	0.544	3544.094	0.2	0.300
Age	-1307.466	0.178	0.214	-1871.639	0.015	0.045	-880.85	0.179	0.269	-794.188	0.013	0.039
Age^2^	28.182	0.389	0.389	43.27	0.091	0.182	21.091	0.341	0.409	20.234	0.06	0.120
Group^*^ Age	2086.407	0.134	0.268	1752.484	0.106	0.159	2072.384	0.03	0.090	547.21	0.224	0.269
Group^*^ Age^2^	-65.477	0.156	0.234	-51.782	0.148	0.178	-62.89	0.046	0.092	-17.332	0.245	0.245

*represents interactions between factors in this table

## Discussion

To our best knowledge, this is the first study on RTT investigating the effect of age on the changes in TIV, cerebral white matter volume, and cortical gray matter volume, especially in younger and older age groups. Although most previous studies have reported about the changes in brain weight and volume in individuals with RTT [6, 9, 20, 37, 40], our investigation is a pioneering study that focused on the correlation between age and brain volume within a larger age range using MRI. We enrolled 28 participants aged 2–27 years who underwent MRI examinations and divided them into younger and older groups for comparison. Individuals in the younger group were in stages 2–4, whereas those in the older group were in stages 3–4. Results demonstrated a significant decrease in the brain volume of different lobes in participants with RTT compared with that in controls. The difference became larger in adulthood when the individuals were in stages 3–4.

The clinical features of RTT are characterized by progressive motor and cognitive deficits [23, 24] and are classified into four stages depending on the clinical manifestations. In our study, the results obtained from RSSS supported the deterioration of clinical severity in the older RTT group when the patients reached the later stage of RTT. *MECP2* controls neuronal development of the brain, regulates neuronal maturation, and even affects neural development and functions in adults [2, 4, 7, 13, 21, 22, 35]. A mouse study demonstrated a higher expression of MECP2 in the adult brain, suggesting that the negative effect of MECP2 dysfunction on the brain is more significant in adulthood when individuals with RTT progressed to stages 3–4 in clinical severity. Neuropathological research has demonstrated some obvious changes in the total brain volume and cerebral size in RTT. Reduced brain size was not attributed to atrophy but to smaller neuronal soma size and dendritic arborizations in the cerebral cortex [3], which may originate from the role and dysfunction of MECP2 in the brain. Nonetheless, there is a paucity of information on the parameters of brain to understand brain development in RTT. Our study demonstrated a decrease in brain volume in most cerebral lobes in participants with RTT compared with that in controls, providing a significant parameter to follow up the development of brain in individuals with RTT.

### Effect of age on changes in brain volume

Our study also demonstrated a distinctive developmental pattern of brain volume in patients with RTT in the younger and older groups. The TIV and cerebral white matter volume significantly increased with age in the control group compared with that in the RTT group. In contrast, there was a distinctive developmental pattern of the cortical gray matter in participants with RTT. Individuals with RTT demonstrated a reduction in cerebral gray matter volume from the very early stage of brain development when there was a deterioration of motor function in stage 2 of RTT and reached a plateau at older age when there was stabilization of motor function in the later stage (Fig. 1). This result indicates that although the expression of MECP2 is lower in the early stage of brain development, it will also affect the early stage of brain development [13, 21], which was rarely discussed in previous studies, and may cause early motor symptoms of stages 1–2 in RTT [38]. The reduction of gray matter volume reached a plateau at older age, which was consistent with worse motor function in the older group at stage 4 of RTT, indicating the crucial role of the higher expression of MECP2 in the adult brain [33, 39].

The intracranial space increases exponentially from early childhood to early adolescence [11], when the expression of MECP2 increases. In our study, the TIV increased from 1200 to 1700 cm^3^ at approximately 15 years of age in participants in the control group. However, the brain volume in participants of the RTT group showed no increase and remained at approximately 1100 cm^3^ in adulthood. This result confirms a significant difference in TIV between the control and RTT groups and may be related to the critical role of MECP2 in neuronal differentiation, terminal maturation, and synaptic maturation, which results in a small head circumference in individuals with RTT [23, 38]. The poor neuronal development with microcephaly may cause poor motor function in later stages of RTT. Our study also revealed a significantly lower cerebral white matter volume in individuals with RTT, and the development of white matter volume in individuals in the control group increased at a faster rate with age than that in individuals with RTT. The lower cerebral white matter volume may be related to the decreased gray matter volume in older age and may also result in poor motor control and clinical severity in the later stage of RTT. This finding had rarely been discussed in previous research [37].

Regarding cortical gray matter development, we found that the volume of cortical gray matter decreased with age in individuals in the control and RTT groups (Fig. 2). It also reached a plateau in adulthood when individuals with RTT are in stages 3–4, when the expression of MECP2 would be higher. This finding is consistent with that reported in previous studies [19, 30] and correlates with the role and expression of MECP2 in different developmental stages. The higher expression of MECP2 in adulthood may be associated with more severe clinical severity, and therefore, leading to advanced stage in RTT.

### Findings on cortical gray matter volume changes

Typically, the gray matter in each cortical region follows a specific developmental pattern, and the volume in each region would reach a peak around a certain age and start decreasing [30]. The present study showed that, in the RTT group, the volume of cortical gray matter decreased with age in all four cortical regions. Remarkably, there was a significant volume decrease in the parietal lobe, with a decrease of 1756 mm^3^ of gray matter volume each year, consistent with a previous study [9].

We conducted further evaluation using one-way ANCOVA, with TIV as a covariate to measure the cortical gray matter volume, to compare the difference between controls and participants with RTT in the younger and older groups. Younger individuals showed reductions in cortical gray matter volume in all four lobes. Older individuals also showed reductions in cortical gray matter volume in all regions, resulting in a greater significant difference between the older control and RTT groups. Furthermore, we compared the age-related change in cortical gray matter volumes compared between the RTT and control groups using a general linear model, which demonstrated a significant difference in bilateral parietal and left occipital lobes. Considering the results from ANCOVA and the general linear model, the cortical gray matter volume reduction was significantly affected by RTT, and the reduction would continue persistently into adulthood.

The parietal lobe is implicated in tactile information processing and sensorimotor integration and may be essential for proper hand functional use [9]. The finding of decreased volume in parietal lobes was also consistent with a recent MRI study in an *Mecp2*-null mouse model [1], showing the decreased brain volume in the somatosensory area. The decreased gray matter volume in parietal lobes with age may explain the pain insensitivity and decreased proprioception with impaired hand functional use in individuals with RTT. Although the previous study demonstrated relative sparing of the occipital lobe compared with other lobes in RTT [9], our study also demonstrated a reduction in the volume of the left occipital lobe with age, consistent with a previous study on visual abnormality in RTT and a recent MRI study using a mouse model [1, 29].

### Effect of MECP2 on brain volume

*MECP2* is a crucial gene that causes RTT [2]. It plays a key role in epigenetic control. Epigenetic control alters gene expression through DNA methylation and histone tail modification and may cause repression of specific gene transcription. *MECP2* was also found to be helpful in regulating and controlling neuronal maturation, dendritic morphology, and synaptic transmission in development in an RTT mouse model [4, 7, 22]. Although MECP2 is predominantly expressed in mature neurons, it is also expressed in the early stage of brain development and therefore affects the early stage of brain development [13, 21], causing the phenotypes in stages 1–2 of RTT. The expression of this protein increases dramatically during gestation and continues till the age of 10 years in the cerebral cortex [41], and it may be higher in adulthood [39]. The distribution of MECP2 in the adult mouse brain varies in different regions. Its expression is higher specifically in the olfactory bulb, cortex, striatum, hippocampus, thalamus, cerebellum, and brain stem. Therefore, with the absence of appropriate MECP2 function and its downstream gene, similar to the brain-derived neurotrophic factor, the regions with high expression of MECP2 will be more severely affected, leading to the delayed onset of clinical presentations, more decrease in brain volume, and also more severe neurological symptoms in adulthood. These data provide vital information on brain development and clinical severity, which may be related to the expression of MECP2 in the brain.

In this study, we detected a significant decrease in cortical gray matter volume in both younger and older individuals with RTT, with more severe reduction in older individuals. Such decrease may be related to the dysfunction of MECP2 and its downstream genes, which may further result in immature brain structure and insufficient maturation of the gray matter even in adults [22, 31, 35]. Because MECP2 expression is higher in adulthood, its dysfunction may result in more impaired motor function and decreased brain volume in older individuals with RTT of stages 3–4. In our study, we found that, among younger participants, the volume of temporal lobes in participants with RTT was not significantly different from that in controls. However, among older participants, the temporal lobes were significantly smaller in volume than those in control participants. This was also consistent with the expression of MECP2 in the brain, with higher expression in the hippocampus causing a more severe decrease in the brain volume of temporal lobes in older individuals.

Our study also demonstrated a significant slow growth of white matter volume in individuals with RTT, which was consistent with a diffusion tensor imaging study [34]. Moreover, we found an increasing difference in white matter volume with age between the control and RTT groups. This result suggests that the dysfunction of MECP2 continues to affect synaptic plasticity and connections in the adult brain of individuals with RTT, resulting in a much significant reduction of brain volume in the later stage of RTT brain and also a deterioration of clinical severity to stages 3–4. Our study emphasizes the important role of MECP2 in both younger and older individuals with RTT, especially in older individuals, and the relationship between the motor function of different stages and brain volume change.

### Limitation

This study has some limitations that should be considered. First, there was no appropriate MRI template available for children; instead, we performed high-resolution imaging and used a created universal template that was described in the methods section. The utilization of the same template may potentially affect the accuracy of the spatial placements when transferring each individual scan. Nevertheless, we performed visual inspection and then manual correction or exclusion for better segmentation. Second, although there were no motion artifacts for older neurotypical individuals, this is a major issue for those with RTT and younger neurotypical individuals. Therefore, we completed our experiments with sedation in participants with RTT and younger neurotypical individuals. There was no related study demonstrating whether the sedation would affect the brain volume. Nonetheless, although we performed sedation in participants with RTT and younger neurotypical individuals, there were some motion artifacts in rare RTT individuals because of difficult sedation, due to which we repeated the experiments several times to obtain better segmentation. There was also no study showing the effect of repeated sedation on brain volume. Third, this study does not have a scope to discuss the complete developmental pattern of the brain as some developmental stages were not investigated (e.g., late childhood/adolescence). Finally, the small sample size and concerns of statistical power were also limitations. Nevertheless, due to the low prevalence of RTT, it is difficult to enroll more cases from only one country, and it is also very difficult to perform brain MRI for adults with RTT with severe disability. Therefore, a further international longitudinal study with more cases from experienced centers is necessary to confirm the age-related change patterns observed in the present cross-sectional study. According to McGraw et al. [35], the importance of *MECP2* is not restricted to early life, and *MECP2* may modulate neural function continuously into adulthood [35]. Therefore, it is critical to examine the effect of RTT across an individual’s life span.

## Conclusion

This is a pioneering MRI study that investigated brain volume development in participants with RTT from early childhood to adulthood and compared the difference in younger age and older age in different stages of RTT. Multiple valuable findings were highlighted in this study as follows: (1) the clinical severity increased with age, and older participants presented similar motor performance as that of younger participants; (2) the TIV and cerebral white matter volume significantly increased with age in typically developed brain compared with that in RTT-affected brain; (3) the gray matter in each cortical region typically follows a specific developmental change and decreases at a certain age. A significant volume decrease with age was found in bilateral parietal lobes and the left occipital lobe of participants with RTT; and (4) the differences in cortical gray matter volume between typically developing brain and RTT-affected brain may tend to continuously increase until adulthood when individuals with RTT reach clinical stage 4.

### Supplementary Information

Supplementary Material 1.

Supplementary Material 2.

Supplementary Material 3.

## Data Availability

Anonymized quantitative data analyzed during the current study is available from the corresponding author upon reasonable request.

## Abbreviations

RTT: Rett syndrome
MECP2: Methyl-CpG-binding protein 2
MRI: Magnetic resonance imaging
MPRAGE: Magnetization-prepared rapid gradient-echo
TR: Repetition time
TE: Echo time
FOV: Field of view
DKT: Desikan–Killiany–Tourville
PDMS-2: Peabody Developmental Motor Scales-second edition
RSBQ: The Rett Syndrome Behavior Questionnaire
RSSS: The Rett Syndrome Severity Scale
ANCOVA: Analysis of covariance
FDR: False discovery rate
TIV: Total intracranial volume
