# Supplementary material for: Developmental change of brain volume in Rett syndrome in Taiwan
Source: J Neurodev Disord. 2024 Jul 3;16:36. doi: 10.1186/s11689-024-09549-6 (PMC11223417; doi:10.1186/s11689-024-09549-6)
Supplement: Supplementary file 1 — Supplementary Material 1. [file 11689_2024_9549_MOESM1_ESM.docx]

Supple Table 1. Group difference compared by independent T- test for total intracranial volume (cm^3^), total cortical gray matter volume (mm^3^), and cerebral white matter volume.

| **Independent Samples Test** | | | | | | |
| --- | --- | --- | --- | --- | --- | --- |
|  | **TIV (cm^3^)** | | **GM (mm^3^)** | | **WM (mm^3^)** | |
| **Group** | **CTL** | **RTT** | **CTL** | **RTT** | **CTL** | **RTT** |
| **Mean±SD** | 1424.42±115.92 | 1142.36±112.17 | 467707.75±39561.12 | 335554.10±61257.42 | 394428.39±69604.03 | 294971.91±42728.98 |
| **P value** | <0.001 | | <0.001 | | <0.001 | |
| TIV: total intracranial volume; GM: cortical gray matter; WM: cerebral white matter; CTL: control group; RTT: Rett syndrome group. | | | | | | |
