# Supplementary material for: Developmental change of brain volume in Rett syndrome in Taiwan
Source: J Neurodev Disord. 2024 Jul 3;16:36. doi: 10.1186/s11689-024-09549-6 (PMC11223417; doi:10.1186/s11689-024-09549-6)
Supplement: Supplementary file 2 — Supplementary Material 2. [file 11689_2024_9549_MOESM2_ESM.docx]

Supple Table 2. Developmental curve for total intracranial volume (cm^3^), total cortical gray matter volume (mm^3^), and cerebral white matter volume.

|  | **Linear regression** | | | | | **Quadratic regression** | | | |  |
| --- | --- | --- | --- | --- | --- | --- | --- | --- | --- | --- |
| **TIV** | **R^2^** |  | **B** | **P** | **R^2^** | |  | **B** | **P** |  |
|  | 0.658 | Intercept | 1168.897 | <0.001 | 0.650 | | Intercept | 1129.002 | <0.001 |  |
|  |  | Group | 163.717 | 0.005 |  | | Group | 217.129 | <0.001 |  |
|  |  | Age | -1.993 | 0.449 |  | | Age | 4.821 | 0.564 |  |
|  |  | Group*Age | 8.316 | 0.022 |  | | Age^2^ | -.212 | 0.465 |  |
|  |  |  |  |  |  | | Group* Age^2^ | 0.242 | 0.049 |  |
| **GM** | **R^2^** |  | **B** | **P** | **R^2^** | |  | **B** | **P** |  |
|  | 0.704 | Intercept | 380932.692 | <0.001 | 0.701 | | Intercept | 378932.405 | <0.001 |  |
|  |  | Group | 115277.516 | <0.001 |  | | Group | 128287.607 | <0.001 |  |
|  |  | Age | -3408.171 | 0.003 |  | | Age | -3554.031 | 0.317 |  |
|  |  | Group*Age | 1441.039 | 0.344 |  | | Age^2^ | 16.466 | 0.893 |  |
|  |  |  |  |  |  | | Group* Age^2^ | 27.080 | 0.598 |  |
| **WM** | **R^2^** |  | **B** | **P** | **R^2^** | |  | **B** | **P** |  |
|  | 0.696 | Intercept | 274040.215 | <0.001 | 0.696 | | Intercept | 235065.596 | <0.001 |  |
|  |  | Group | 25529.406 | 0.262 |  | | Group | 54395.475 | 0.002 |  |
|  |  | Age | 1572.081 | 0.137 |  | | Age | 8866.304 | 0.009 |  |
|  |  | Group*Age | 4961.361 | 0.001 |  | | Age^2^ | -242.853 | 0.037 |  |
|  |  |  |  |  |  | | Group* Age^2^ | 156.841 | 0.002 |  |
| TIV: total intracranial volume; GM: cortical gray matter; WM: cerebral white matter; CTL: control group; RTT: Rett syndrome group. | | | | | | | | | | |
