# Supplementary material for: Developmental change of brain volume in Rett syndrome in Taiwan
Source: J Neurodev Disord. 2024 Jul 3;16:36. doi: 10.1186/s11689-024-09549-6 (PMC11223417; doi:10.1186/s11689-024-09549-6)
Supplement: Supplementary file 3 — Supplementary Material 3. [file 11689_2024_9549_MOESM3_ESM.docx]

Supple Table 3. Regional difference of cortical gray matter volume between Rett syndrome and healthy control groups. The statistics were done to compare the Rett syndrome and health control groups in different lobes.

| **Independent Samples Test** | | | | | | | | |
| --- | --- | --- | --- | --- | --- | --- | --- | --- |
|  | **Control**  **N = 32** | | **Rett Syndrome**  **N = 28** | |  |  | **95% Confidence Interval of the Difference** | |
|  | M | SD | M | SD | t | Sig. (2-tailed) | Lower | Upper |
| Age | 14.52 | 8.23 | 13.31 | 8.03 | -.572 | 0.569 | -5.42 | 3.0086973 |
| **Right Hemisphere** | | | | | | | | |
| Frontal lobe | 86355.03 | 8204.81 | 61920.89 | 11071.31 | -9.789 | <0.001 | -29430.59 | -19437.69 |
| Parietal lobe | 67643.78 | 6686.86 | 47545.71 | 9726.41 | -9.423 | <0.001 | -24367.61 | -15828.52 |
| Temporal lobe | 53836.81 | 5103.90 | 38865.46 | 7400.76 | -9.215 | <0.001 | -18223.60 | -11719.10 |
| Occipital lobe | 26927.53 | 2687.97 | 20486.07 | 3754.82 | -7.710 | <0.001 | -8113.94 | -4768.98 |
| **Left Hemisphere** | | | | | | | | |
| Frontal lobe | 86335.88 | 8218.81 | 61746.11 | 11121.91 | -9.817 | <0.001 | -29603.53 | -19576.01 |
| Parietal lobe | 65690.84 | 6463.84 | 46473.68 | 9802.98 | -9.068 | <0.001 | -23459.24 | -14975.09 |
| Temporal lobe | 54311.22 | 5282.69 | 38399.32 | 7567.45 | -9.536 | <0.001 | -19251.82 | -12571.978 |
| Occipital lobe | 26606.66 | 2908.85 | 20116.86 | 3772.11 | -7.512 | <0.001 | -8219.17 | -4760.427 |
